# A new analysis approach for single nephron GFR in intravital microscopy of mice

**DOI:** 10.12688/f1000research.26888.3

**Published:** 2021-06-10

**Authors:** Friederike Kessel, Hannah Kröger, Michael Gerlach, Jan Sradnick, Florian Gembardt, Vladimir Todorov, Christian Hugo

**Affiliations:** 1 Experimental Nephrology and Division of Nephrology, Department of Internal Medicine III, University Hospital Carl Gustav Carus at the Technische Universität Dresden, Fetscherstraße 74, Dresden, 01307, Germany; 2 Core Facility Cellular Imaging (CFCI), University Hospital Carl Gustav Carus at the Technische Universität Dresden, Fetscherstraße 74, Dresden, 01307, Germany

**Keywords:** Intravital Microscopy, 2-Photon Microscopy, Kidney, Single Nephron GFR, ImageJ, R

## Abstract

**Background: **Intravital microscopy is an emerging technique in life science with applications in kidney research. Longitudinal observation of (patho-)physiological processes in living mice is possible in the smallest functional unit of the kidney, a single nephron (sn). In particular, effects on glomerular filtration rate (GFR) - a key parameter of renal function - can be assessed.

**Methods:** After intravenous injection of a freely filtered, non-resorbable, fluorescent dye in C57BL/6 mice, a time series was captured by multiphoton microsopy. Filtration was observed from the glomerular capillaries to the proximal tubule (PT) and the tubular signal intensity shift was analyzed to calculate the snGFR.

**Results:** Previously described methods for snGFR analysis relied on two manually defined measurement points in the PT and the tubular volume was merely estimated in 2D images. We present an extended image processing workflow by adding continuous measurement of intensity along the PT in every frame of the time series using ImageJ. Automatic modelling of actual PT volume in a 3D dataset replaced 2D volume estimation. Subsequent data analysis in R, with a calculation of intensity shifts in every frame and normalization against tubular volume, allowed exact assessment of snGFR by linear regression. Repeated analysis of image data obtained in healthy mice showed a striking increase of reproducibility by reduction of user interaction.

**Conclusions:** These improvements in image processing and data analysis maximize the reliability of a sophisticated intravital microscopy technique for the precise assessment of snGFR, a highly relevant predictor of kidney function.

## Introduction

Glomerular filtration rate (GFR) is a key parameter of kidney function and deviations from normal GFR are a hallmark of renal diseases ^1, 2^. GFR describes the filtration of substances from blood in the glomerular capillaries, to the primary urine in the tubular system of the kidney. Therefore, changes in GFR serve to monitor disease progression ^1, 2^. GFR is also measured in animal models to study effects of pharmacological intervention on kidney function ^3^. Advances in intravital imaging and multiphoton microscopy allow repetitive assessment of GFR and morphological changes in the smallest functional unit of the kidney – the nephron ^3– 5^. Longitudinal imaging of single nephrons (sn) enable direct correlation of structural and functional data ^3– 5^.

After intravenous injection of the freely filtered, non-resorbable, fluorescent dye LuciferYellow (LY), a time series was captured by multiphoton microsopy. Filtration was observed from the glomerular capillaries to the proximal tubule (PT) and the tubular signal intensity shift is analyzed to calculate the filtration rate. Translated to an image processing task, this can be generalized as the flow rate in a tube. Previous methods for this analysis ^3, 4^ relied on two manually annotated measurement points in the PT and stereotypic estimation of PT volume in 2D images. Since results we obtained with this approach were highly variable, we expanded the analysis of image data via 3D modelling with open source software, to increase overall reproducibility and reliability of the analysis when comparing renal function of different experimental groups.

## Methods

### Animal experiments

Animal experiments were performed in accordance with the Federation of European Laboratory Animal Science Associations (FELASA) Guidelines for the Care and Use of Laboratory Animals and the Federal Law on the Use of Experimental Animals in Germany and approved by the ethical review committee at the Landesdirektion Sachsen (license DD-24.1-5131/338/37). For microscopy, male, 10–12 week old C57BL/6 mice were prepared as previously described ^5, 6^. In brief, a titanium abdominal imaging window (AIW) covered with a coverslip is surgically implanted above the kidney. The kidney is glued to the coverslip with cyanoacrylate glue before securing the AIW by tightening the skin in the AIW groove. Microscopy was performed one day after AIW implantation.

A custom-built temporary intravenous catheter (polyethylene tubing #587360 by Science Products GmbH with 0.3×12mm needle) was placed in the lateral tail vein. Fluorescent dyes were administered into the tail vein prior (Hoechst, AngioSpark) or during (LuciferYellow) microscopy (detailed information in Table 1).

**Table 1.** Dyes.

Dye	Order Number	Supplier	Purpose	Application details	Channel	Exitation	Acquisition
AngioSPARK 680	NEV10149	PerkinElmer	Vessel dye	30 µl	3	860 nm	685–695 nm
Hoechst 33342	H3570	Thermo Fisher	Nuclear dye	50 µl (2 mg/ml)	4	860 nm	415–474nm
Lucifer Yellow CH dilithium salt	L0259- 25MG	Sigma Aldrich	Freely filtered flourescent dye	20 µl via syringe pump in 1 s (5 mg/ml)	2	860 nm	500–550nm

All efforts were made to ameliorate harm to animals. Imaging (including injections of the fluorescent dyes) and the implantation is done under isoflurane anaesthesia. The image data of the five animals presented for the comparison of the extended workflow with the previous workflow in this manuscript were generated previously as part of an independent experiment (license DD-24.1-5131/338/37).

### Microscopy

Imaging was performed on an upright Leica SP8 multiphoton laser scanning microscope at the Core Facility Cellular Imaging. Settings for signal acquisition are summarized in Table 2.

**Table 2.** Image acquisition settings.

Dye	Exitation	Objective	Resolution	Detection
AngioSPARK 680	860 nm, Chameleon II (Coherent)	40x 1.1 NA water immersion objective	Pixel size: 0.8513 µm frame rate (time series): 6 fps Voxel depth (z-stack): 1 µm	685-695 nm, HyD detector (Leica)
Hoechst 33342				415-474nm, PMT detector (Leica)
Lucifer Yellow CH dilithium salt				500-550nm, HyD detector (Leica)

### Image and data analysis

Image processing and analysis was done in ImageJ ^7– 9^ (1.53c) with 3D ImageJ Suite ^10^ and Bio-Formats ^11^ for the use of 3D image processing plugins and the Bio-Formats Importer. Data analysis was performed in R ^12^ (4.0.2), with RStudio ^13^ (1.2.5033) including ggplot2 ^14^ (including dependencies) installed as additional library. The script executed the ImageJ macro from command line and subsequently analyzed and visualized the results. A detailed description of the algorithm is associated with the scripts on GitHub ^15^.

The line region of interest (ROI) set for the extended workflow to manually define direction and position of the proximal tubule (PT) was also used to determine the two measuring points (beginning and end of line) for analysis of image material based on the previously described approach ^3, 4^. Tubular diameter was calculated as the mean of five manually measured diameters.

## Results

**Figure 1.**
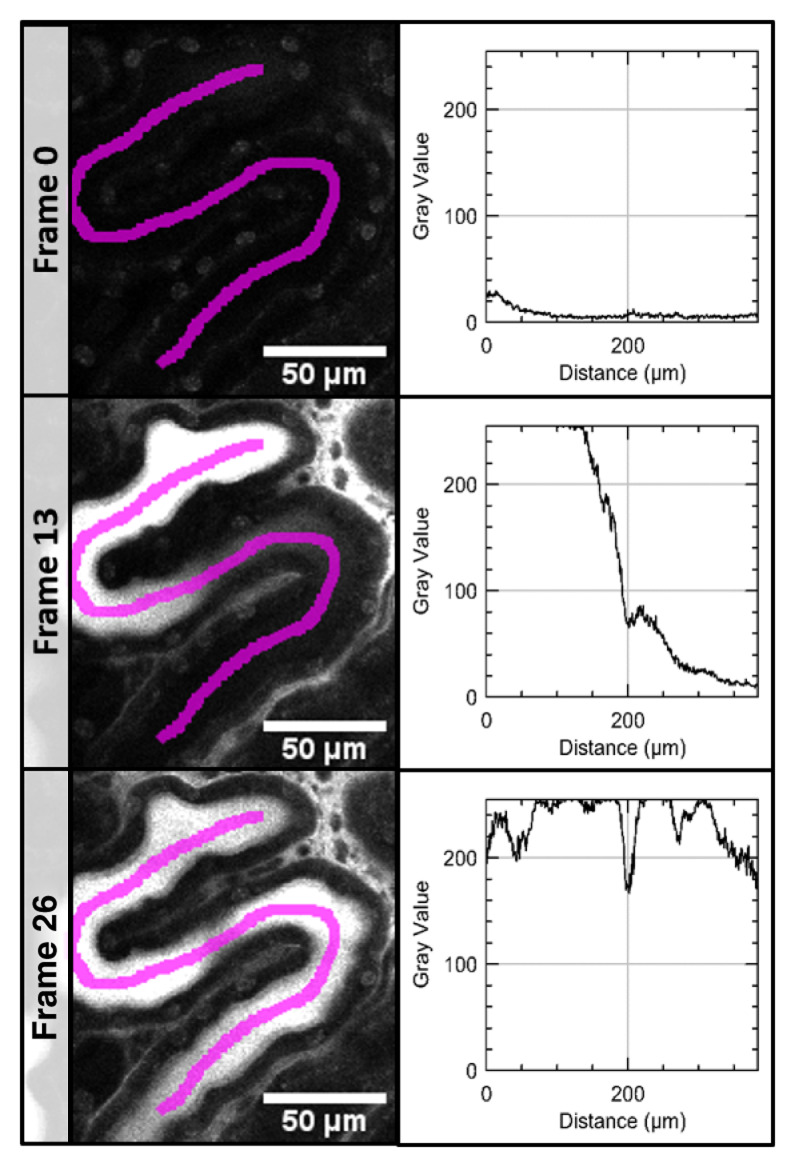
Measurement of signal intensity in a time series of the proximale tubule (PT). Signal intensity of LuciferYellow (LY) was measured along a line region of interest (ROI, magenta) in every frame (here only frame 0 - before LY injection, frame 13 and 26 are shown on the left). For visualization of the resulting data, the signal intensity along the ROI is plotted for these sample frames on the right. As the LY moves through the PT, the measured signal intensity shifts as well.

In the time series acquired after application of LY, a line ROI was set to manually define the position and direction of the measurement. Along this ROI, x-y plots measured the dye intensity in the PT in every frame ( Figure 1) and numerical results were saved.

**Figure 2.**
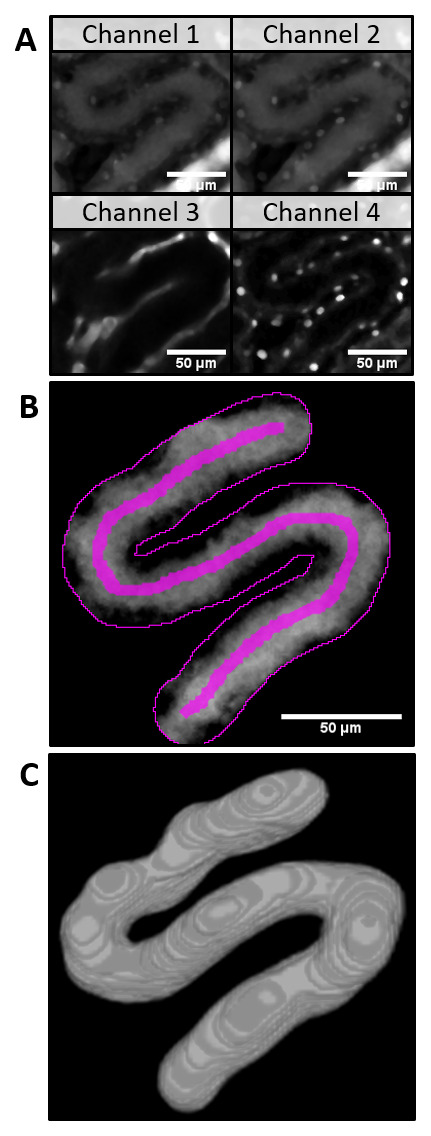
Automatic 3D modelling of tubular volume in a z-stack of the proximal tubule (PT). **A**) After applying a 3D median filter, the channel 3 and channel 4 z-stacks were subtracted from channel 2 to eliminate spectral bleedthrough artifacts ( **B**). The proximal tubule (PT) was segmented with the help of a 3D watershed (3D model of the resulting z-stack, **C**).

**Figure 3.**
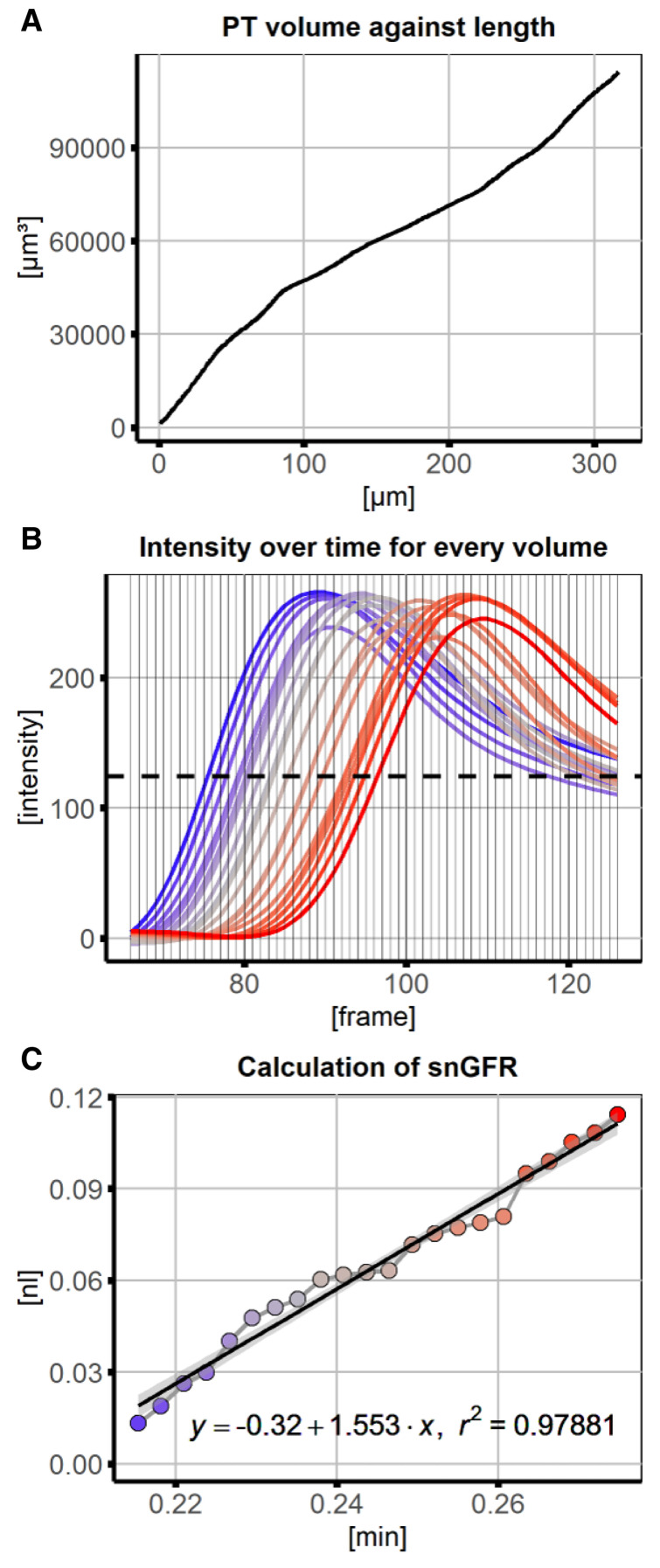
Data analysis and linear regression of signal volume against time for calcuation of glomerular filtration rate (GFR). **A**) For every position along the line region of interest (ROI), the cumulative volume was measured, providing a conversion of position to volume. **B**) Numerical data underlying the x-y plots was saved and used to subsequently plot changes of signal intensity over time for every position (and converted to cumulative volume) along the line ROI. The dashed line represents the threshold value at which the corresponding volume of the proximal tubule (PT) was approximated for every frame. **C**) Using linear regression the snGFR could be calculated as the volume with the intensity threshold at the frames of interest (after conversion from µm ^3^ per frame to nl per minute). Regression line is displayed with 95% confidence interval. The colour codes for the position along the PT (blue – beginning, red – end).

For the automatic 3D modelling of PT volume the z-stack of the same field of view was acquired. Additional channels (Ch3: AngioSpark - vessels, Ch4: Hoechst - nuclei, Figure 2A) were subtracted plane by plane from Ch2 (target channel, LY intensity) to remove spectral bleed-through artifacts ( Figure 2B). With the 3D watershed, the PT was segmented ( Figure 2C, 3D-model) and saved for visual verification. The cumulative PT volume was measured over the distance along the ROI and plotted in subsequent data analysis ( Figure 3A). The position is now recalculated to the cumulative PT volume at each point along the ROI. From intensity measurements a threshold intensity was set to the turning point of fluorescence intensity over time at every volume (maximum slope, Figure 3B). The volume with this intensity was approximated in each frame and used for linear regression ( Figure 3C, intersect of horizontal threshold at every frame with intensity curves). The slope of the regression line equals the snGFR after conversion of µm³ per frame to nl per minute. Together with information about PT length, PT volume and R-squared the results were summarized and saved in a data table.

**Figure 4.**
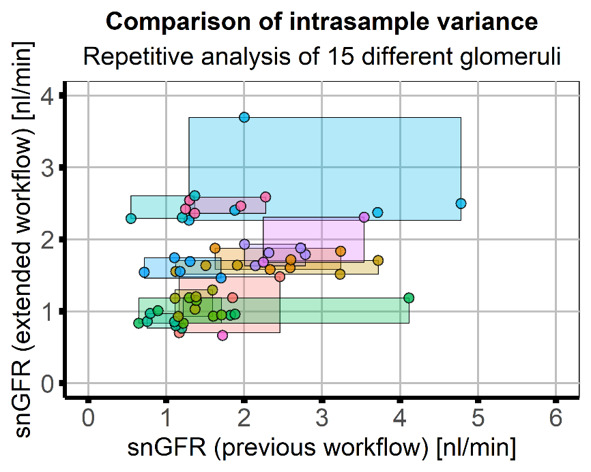
Application and comparison of the workflows in image data of healthy mice. Image data of healthy mice (five animals, 15 glomeruli) was analysed five times by the same researcher using the previous and the extended workflow. Scatter plot of results of the previous (x-axis) and extended workflow (y-axis) with rectangles used to indicate the range of results obtained in one glomerulus. Colours indicate data obtained from individual glomeruli. Intrasample variance with the extended workflow (variance along the y-axis, mean CV=10.35%) was smaller than with the previous workflow (variance along the x-axis, mean CV=38.75%). Both analysis workflows showed similar results (mean snGFR, previous workflow: 1.71±0.91, extended workflow: 1.70±0.78) and a two-sample Kolmogorov-Smirnov test of both result vectors showed that the distributions were not statistically different (p=0.4662).

Repeated analysis (five times) of 15 individual glomeruli by the same researcher showed that results obtained with the presented workflow had higher consistency (lower intrasample variance, CV=10.35%) compared to the previous approach (CV=38.75%, Figure 4). Due to the high variance with the previous approach a direct correlation of the workflows was not possible; however, the final result - the mean snGFR - was comparable (previous workflow: 1.71±0.91, extended workflow: 1.70±0.78) and a two-sample Kolmogorov-Smirnof test of both result vectors showed that the distributions were not statistically different (p=0.4662). Numerical results of the repeated analysis with both workflows are listed in Table 3.

**Table 3.** Numerical data of repeated analysis with the previous and the extended workflow.

	Previous Workflow			Extended Workflow		
	Mean	Standard deviation (SD)	Relative SD [%]	Mean	SD	Relative SD [%]
Dataset 1	1.783	1.057	59.254	1.724	0.132	7.675
Dataset 2	2.476	0.581	23.451	1.611	0.075	4.676
Dataset 3	2.296	1.123	48.900	2.587	0.125	4.842
Dataset 4	0.606	0.211	34.862	1.128	0.147	13.012
Dataset 5	1.441	0.207	14.355	1.012	0.151	14.968
Dataset 6	1.871	1.367	73.039	0.987	0.127	12.873
Dataset 7	0.995	0.204	20.535	0.851	0.077	9.078
Dataset 8	1.039	0.433	41.661	2.600	0.304	11.708
Dataset 9	2.732	1.456	53.306	2.648	0.593	22.375
Dataset 10	1.200	0.356	29.644	1.600	0.116	7.254
Dataset 11	2.393	0.347	14.505	1.811	0.113	6.265
Dataset 12	1.359	0.666	48.997	2.987	0.124	4.139
Dataset 13	1.628	0.460	28.242	2.477	0.091	3.665
Dataset 14	3.746	1.603	42.805	0.717	0.176	24.566
Dataset 15	0.176	0.084	47.642	0.814	0.067	8.225

## Conclusions

The progressive development of microscopy techniques like measurement of snGFR in experimental animals needs to be accompanied by improvements in analysis algorithms to use their full potential. In this manuscript we present a workflow by extending an existing analysis method via 3D modelling, for increased reproducibility, accuracy, but also transparency in the measurement of snGFR. By reducing user interaction, intrasample variance was markedly improved.

Additionally, the automatically saved user input and intermediate results (z-stack of watershed of PT as shown in Figure 2C and graphs in Figure 4) for every analyzed dataset provide full possibility to verify every analysis step. These results can be used to objectively evaluate the measurement. Although the snGFR in this manuscript was very low for healthy animals compared to previously published values ^3^, the range was comparable in both methods and not an artifact produced by the workflow but more likely caused by the general experimental setup.

Taken together, this workflow extension contributes to an overall improvement of the interpretation of snGFR measurements. Applied to experimental data this can cumulate in a higher power to detect statistically significant differences between experimental groups and even decrease the necessary sample size, thus having an impact on animal welfare.

## Data availability

### Underlying data

Zenodo: Sample dataset - cont-3D-snGFR. https://doi.org/10.5281/zenodo.4275596 ^16^.

This project contains the following underlying data:

- Sample_Dataset_cont-3D-snGFR.lif (Sample file with time series and z-stack of three different glomeruli after injection of LuciferYellow for the analysis of single nephron GFR)
- Results.zip (Sample file for the selection (ROI sets) of the proximal tubulus in the sample dataset, including the resulting measurements (text files) in the time series and 3D modelling of the proximal tubules (tiff files))
- Graphs_2020-09-30.zip (Intermediate results and graphs (png files) as obtained from the sample dataset with selections and measurement data in the results file)
- 2020-09-30-Result_summary.txt (Final summary (text file) of calculated single nephron GFR for the three sample glomeruli based on selections from the results file)
- Dataset1.lif (Image data used for the comparison of previous and extended workflow in Figure 4, includes 15 time series and the corresponding z-stacks)

Data are available under the terms of the Creative Commons Attribution 4.0 International license (CC-BY 4.0).

## Software availability

Source code available from: https://github.com/NephrologieDresden/cont-3D-snGFR

Archived source code at time of publication: https://doi.org/10.5281/zenodo.4642427 ^15^.

License: GNU General Public License v3.0
