# Supplementary material for: A new analysis approach for single nephron GFR in intravital microscopy of mice
Source: F1000Res. 2021 Jun 10;9:1372. Originally published 2020 Nov 26. [Version 3] doi: 10.12688/f1000research.26888.3 (PMC8210690; doi:10.12688/f1000research.26888.3)
Supplement: Peer review history

# Peer review: Reviewer response for version 3

**DOI:** 10.5256/f1000research.57648.r87230

**Published:** 2021-06-16

**Authors:** Anna Schueth

**Affiliations:** 1 Department of Cognitive Neuroscience, Faculty of Psychology & Neuroscience, Maastricht University, Maastricht, The Netherlands

I have no further comments to make.

Is the work clearly and accurately presented and does it cite the current literature?

No

If applicable, is the statistical analysis and its interpretation appropriate?

I cannot comment. A qualified statistician is required.

Are all the source data underlying the results available to ensure full reproducibility?

Yes

Is the study design appropriate and is the work technically sound?

Yes

Are the conclusions drawn adequately supported by the results?

Yes

Are sufficient details of methods and analysis provided to allow replication by others?

Yes

Reviewer Expertise:

Multiphoton microscopy and Light-sheet microscopy with application to mouse modes.

I confirm that I have read this submission and believe that I have an appropriate level of expertise to confirm that it is of an acceptable scientific standard.

# Peer review: Reviewer response for version 2

**DOI:** 10.5256/f1000research.55759.r86304

**Published:** 2021-06-07

**Authors:** Anna Schueth

**Affiliations:** 1 Department of Cognitive Neuroscience, Faculty of Psychology & Neuroscience, Maastricht University, Maastricht, The Netherlands

Here the authors are presenting an intravital multiphoton microscopy study to study the single nephron (sn) and glomerular filtration rate (GFR) in mice. In the results section the authors describe an extended image processing workflow with ImageJ/Fiji and "R". Conclusively, this study shows an improved manner for image processing in order to study and analyse snGFR. The study seems technically sound to me. 

 However, Figure 1 is to my opinion not optimal. If separated from the main manuscript text, it is difficult to understand the presented results in this figure. Therefore, I would advise the authors to revise the figure description.

Is the work clearly and accurately presented and does it cite the current literature?

No

If applicable, is the statistical analysis and its interpretation appropriate?

I cannot comment. A qualified statistician is required.

Are all the source data underlying the results available to ensure full reproducibility?

Yes

Is the study design appropriate and is the work technically sound?

Yes

Are the conclusions drawn adequately supported by the results?

Yes

Are sufficient details of methods and analysis provided to allow replication by others?

Yes

Reviewer Expertise:

Multiphoton microscopy and Light-sheet microscopy with application to mouse modes.

I confirm that I have read this submission and believe that I have an appropriate level of expertise to confirm that it is of an acceptable scientific standard, however I have significant reservations, as outlined above.

# Response

**Published:** 2021-06-08

**Authors:** Friederike Kessel

**Affiliations:** University Hospital Carl Gustav Carus Dresden, Germany

We thank Dr. Schüth for their reviewer report and the helpful remarks on Figure 1 and the insufficient figure captions. We agree that without the context of the entire article, the caption should contain additional information.

 We address this issue in a new version of the manuscript and hope Figure 1 is now more understandable.

# Peer review: Reviewer response for version 2

**DOI:** 10.5256/f1000research.55759.r84810

**Published:** 2021-05-18

**Authors:** Georgina Gyarmati

**Affiliations:** 1 Departments of Physiology and Neuroscience, and Medicine, Zilkha Neurogenetic Institute, University of Southern California, Los Angeles, Los Angeles, CA, USA

Since multiphoton microscopy and other imaging approaches have become more commonly used technologies, the need for standardized and reproducible image analysis methods are much needed. Therefore, the authors' work is timely and important. The authors in this paper addressed the intra-observer variability of single nephron glomerular filtration rate (SNGFR) measurement and provided an extended workflow for image processing and data analysis to maximize reproducibility. The application of the extended workflow resulted in reduced variability of measurements, however, no significant difference was found between results obtained with the conventional and the new method.

 The article is based on a false claim and hypothesis that the current published methods for SNGFR measurement is unreliable and produce high variability. Therefore, its scientific validity is questionable. There seems to be one major and one minor issue with this work. The major issue concerns the unclear overall significance of this technical advance because it provides only a minor incremental advance in the field. The claim that the conventional workflow does not provide reproducible and reliable results is not valid. There are many advantages of the conventional manual analysis method and when performed correctly by the published methods and meticulous observer the results are highly reproducible and have low variability. It is puzzling how the application of the conventional and new approach can produce over 5-fold differences in results from the same sample (Table 3). In my opinion, there are more stressing issues with the measurement of SNGFR, that would warrant the development of such advanced analytical tools. For example, SNGFR is not constant over time as compared to global GFR due to vasomotor (myogenic) tone and tubuloglomerular feedback (TGF). This results in significant alterations in GFR on the single nephron level by every 5-10 seconds (myogenic) or 30-50 seconds (TGF). Therefore, this is a more important issue in SNGFR variability than intra-observer variability in the analysis of a single timepoint SNGFR measurement.

 The minor issue is the quality of the preparations shown in the supplement material as previously raised by Dr. Molitoris. There seems to be a significant amount of blebs and cell debris flowing in many tubular segments. All numerical SNGFR values shown in table 3 are below the physiological range. These suggest that the animals were not in physiological healthy conditions. Using these datasets for the current analytic development work is not optimal.

Is the work clearly and accurately presented and does it cite the current literature?

Yes

If applicable, is the statistical analysis and its interpretation appropriate?

Partly

Are all the source data underlying the results available to ensure full reproducibility?

Yes

Is the study design appropriate and is the work technically sound?

Partly

Are the conclusions drawn adequately supported by the results?

No

Are sufficient details of methods and analysis provided to allow replication by others?

Yes

Reviewer Expertise:

Renal pathophysiology, intravital imaging

I confirm that I have read this submission and believe that I have an appropriate level of expertise to state that I do not consider it to be of an acceptable scientific standard, for reasons outlined above.

# Peer review: Reviewer response for version 2

**DOI:** 10.5256/f1000research.55759.r83125

**Published:** 2021-04-23

**Authors:** Christopher Schmied

**Affiliations:** 1 FMP Berlin, Berlin, Germany

The authors addressed my raised concerns and significantly improved the documentation by supplying a good manual for the workflow. This manual now addresses the important prerequisites of the workflow, how to install any dependencies and how to use the workflow effectively. This should enable users to easily pick up the analysis and sort out any usage problems that might arise. Particularly the description of the results and intermediate files now contain very good comments to interpret and troubleshoot the analysis. Well done!

 Thank you for also clarifying some parts of the manuscript and figures as well as providing further citations.

Is the work clearly and accurately presented and does it cite the current literature?

Partly

If applicable, is the statistical analysis and its interpretation appropriate?

Yes

Are all the source data underlying the results available to ensure full reproducibility?

Partly

Is the study design appropriate and is the work technically sound?

Yes

Are the conclusions drawn adequately supported by the results?

Yes

Are sufficient details of methods and analysis provided to allow replication by others?

No

Reviewer Expertise:

Bioimage analysis, Computer vision, Data science

I confirm that I have read this submission and believe that I have an appropriate level of expertise to confirm that it is of an acceptable scientific standard.

# Peer review: Reviewer response for version 1

**DOI:** 10.5256/f1000research.29693.r76548

**Published:** 2021-01-28

**Authors:** Christopher Schmied

**Affiliations:** 1 FMP Berlin, Berlin, Germany

The authors describe a semi-automated analysis for measuring single nephron glomerular filtration rate (snGFR). An important parameter for assessing renal function. Intravital microscopy was used to record the filtration of a fluorescent dye along glomerular vessels to the proximal tubule. The aim of the analysis is to measure the flow rate. To achieve this the user first sets a line ROI to determine the position and direction for the measurement of the intensity change across the time course. Then the entire 3D volume is segmented in a separate z-stack. The analysis is smart and tries to use all available information in their experimental setup to ensure a robust analysis. The generation of the 3D volume based on a 3D watershed uses the information from different channels to ensure a robust segmentation. Using a regression analysis makes sure that not only 2 points alone will contribute to the final measurement of the snGFR. I think this is a good image and data analysis approach to reduce measurement variability and increase statistical power.

 The workflow runs as R script that also calls a Fiji macro. The interaction runs via sequential GUI prompts. This increases the ease of use. 

 The article is, in general, well written and contains most of the important information to understand the method and how it compares to previously used methods. The detailed description of the algorithm is sometimes a bit confusing but one can understand the rational of the authors. My main problem is with the lack of documentation that allows one to access and implement the tools. Here are my points for revision: 

**MAJOR:** The algorithm makes sense. The description in the text and figure legends is however a bit hard to understand.

 This sentence is particularly unclear: " *The position with this intensity was approximated in each frame and used for linear regression (Figure 3C)*". I guess what the authors wanted to express is that the volume was approximated at this position. Then the approximated volume was plotted over time and based on that linear regression was performed?

**Figure 3 B&C and their legends are rather confusing:**

 Figure 3B mixes in the volume of the segmentation, although this is not the message of the figure (Intensity and computation of threshold on slope). The color code of Figure 3B&C corresponds I guess to the positions? This is not explained anywhere. In Figure 3A first µm³ is used and then in Figure 3C nl?

**MAJOR: **I downloaded the material and it took me about 4 tries to get the scripts to work correctly. Here are the key impediments: 

1. Does not execute on ubuntu 20.04: the R script uses functions that only work under Windows. This limitation needs to be explicitly stated. This also abolishes the advantage of cross platform tools such as Fiji and R. 
2. The 3D watershed is not explicitly stated as dependency of Fiji. It needs to be clearly stated what needs to be installed and how. 
3. The direction of the ROI is important but this was not clear from the documentation. 

**MAJOR:** The Documentation word file provided is not helpful for actually using the scripts. It rather contains a code documentation that has some directions of using the program included. People with little expertise have no clear guidance for the usage and the important settings of the usage are entirely lost in all the detail. 

1. The usage needs to be documented separately from the code. 
2. The actual interaction with the program needs to be documented also via screenshots.
3. The important settings need to be explained clearly and in sufficient detail. 

**MAJOR:** That one needs to draw the ROI in the direction of the wave was not really obviously documented or it got lost in the complexity of the code documentation. Please use screenshots or describe with words. 

**MAJOR: **How the data needs to be acquired and structured for this workflow to function is not explained anywhere. The prompt for selecting a corresponding z-stack made initially zero sense. Since it was not clear that the .lif file must contain the multichannel time series AND the z-stack. Are the channels settings hard coded then? If the analysis is inflexible in its data input (which can be ok), it needs to be mention explicitly as an important prerequisite.

**MAJOR: **Reproducing the workflow using the provided .lif file resulted mostly in snGFR that were in a similar range. But still off. Maybe drawing the ROIs seems to be still an important source of variability. It would be good if there would be an easy way to load and visualize the ROIs provided by the authors. This shows easily how the authors intend users to set ROIs. One can load them via the ROI Manager during the GUI interactions but this produces an error later on: 

 Composite selections cannot be converted to lines. in line 520: 

*(called from line 193)*

*run ( "Area to Line" <)>*

 Maybe it would also be good to document in words along example screenshots how one best should set the ROI.

**MAJOR: **I am not in the kidney field. Maybe certain statements are common knowledge there and it is practice not to cite them. But the following statements in the introduction would strike me as requiring citations: 

- *"Therefore, changes in GFR serve to monitor disease progression."*
- *"GFR is also measured in animal models to study effects of pharmacological intervention on kidney function." *
- *"Advances in intravital imaging and multiphoton microscopy allow repetitive assessment of GFR and morphological changes in the smallest functional unit of the kidney – the nephron."*
- *"Longitudinal imaging of single nephrons (sn) enable direct correlation of structural and functional data."*
- *"Since results we obtained with this approach were highly variable." *

**MINOR: **All the result files produced by the workflow and how to interpret them and recognize issues are not described anywhere in the documentation. 

**MINOR:** It would be nice, at least in the documentation, to layout graphically the flow of the workflow.

**MINOR: **Please include a brief description of the usage in the README. What needs to be installed (also the 3D watershed update site) and how to run the workflow. Also any important prerequisites should be mentioned there as well. 

**MINOR: **Selecting the "executable Fiji file" needs to be described better and documented with a screenshot. Users that have Fiji preinstalled or rarely use Fiji will not know this. 

**MINOR:** Figure 4 is hard to interpret and one cannot easily compare own results for reproducing the workflow. The result of the automatic analysis are included as an extra file. It would be nice to have the results of this automatic and manual analysis available as a table for a direct comparison. 

**MINOR:** I miss an explicit point of contact or means of support for any users such as github, forum or Email.

Is the work clearly and accurately presented and does it cite the current literature?

Partly

If applicable, is the statistical analysis and its interpretation appropriate?

Yes

Are all the source data underlying the results available to ensure full reproducibility?

Partly

Is the study design appropriate and is the work technically sound?

Yes

Are the conclusions drawn adequately supported by the results?

Yes

Are sufficient details of methods and analysis provided to allow replication by others?

No

Reviewer Expertise:

Bioimage analysis, Computer vision, Data science

I confirm that I have read this submission and believe that I have an appropriate level of expertise to confirm that it is of an acceptable scientific standard, however I have significant reservations, as outlined above.

# Response

**Published:** 2021-03-28

**Authors:** Friederike Kessel

**Affiliations:** University Hospital Carl Gustav Carus Dresden, Germany

First of all we thank the reviewer for the extensive report and constructive feedback. We agree that a lack of documentation, guidance and also support information notably impaired the usability of the workflow. In this context, the patience of the reviewer to implement the algorithm is highly appreciated.

 Since most of the remarks were directly linked with lack of documentation, we uploaded detailed instructions to the GitHub repository and updated the associated release on Zenodo. This documentation now includes:

- A paragraph on the structure of the raw data
- System requirements (operating system)
- Software requirements (ImageJ, including update sites, R and RStudio with additional libraries)
- Instructions on how to run the workflow with screenshots
  - Explicitly pointing out the importance of the direction of the line ROI
  - With additional screenshots of example line ROIs for the images included in the sample dataset
- Information on all output files (ROI sets, result files, graphs)
- Suggestions for data interpretation and troubleshooting
- Contact information

Since this analysis was only recently developed and experiences when applying it to different image data are still limited we are determined to continuously expand the documentation and troubleshooting suggestions. We recognize that there is also room for improvement for the programming itself, regarding the limitation to Windows and hard-coded requirements of the raw data. We plan to support the gradual expansion of the workflow to be more adaptable – and applicable – in the future (as mentioned in the documentation).

 Since the reviewer pointed out that some of the descriptions in the manuscript and figure legends were hard to understand, we rephrased some points. We hope it is now more understandable.

- Description of Figure 3C: Approximation of the volume for every position and plotting against time for linear regression
- Legends for Figures B and C: Colour code
- Conversion of units: µm³ to nl in Figure 3C

We also included the table with the numerical results as shown in Figure 4.

 Finally, the statements on GFR and methods in intravital microscopy in the introduction can be supported with references that were already used in other contexts in the manuscript. Therefore, we additionally refer to them in the introduction.

# Peer review: Reviewer response for version 1

**DOI:** 10.5256/f1000research.29693.r75572

**Published:** 2020-12-16

**Authors:** Bruce Molitoris

**Affiliations:** 1 Department of Medicine, Indiana University School of Medicine, Indianapolis, USA

Measurement of SNGFR is an important undertaking and adding a 3D component to tubular volume is interesting. However, viewing glomeruli in mice of 10-12 weeks is not possible without significant invasive proceedures. Ureteral obstruction has been used by some, but this author uses removal of 1 mm of cortical tissue to get down to cortical glomeruli. They do not say this but reference an existing paper. Since you can only see up to 100 microns with the 2-photon scope with high resolution, it is difficult to imaging tissue injury is not altering function of the glomerulus and tubules. This has to be discussed and if controls are available they should be mentioned. In the referenced paper the sieving Coefficient for albumin was very high and likely due to tissue injury. No glomerulus is shown in the present work and yet the authors indicate they followed from glom and then along the tubule. Were they measuring flow in S1 or S2 segments as flow would vary due to reabsorption?

 Also, the subtraction of 3D volumes from each other for background subtraction is not recommended. It is best to subtract each individual plane from the corresponding channels.

 It would also be helpful if they put figure 4 data into a table for easier and direct comparison.

Is the work clearly and accurately presented and does it cite the current literature?

Partly

If applicable, is the statistical analysis and its interpretation appropriate?

Yes

Are all the source data underlying the results available to ensure full reproducibility?

No source data required

Is the study design appropriate and is the work technically sound?

Partly

Are the conclusions drawn adequately supported by the results?

Partly

Are sufficient details of methods and analysis provided to allow replication by others?

No

Reviewer Expertise:

Nephrology, imaging

I confirm that I have read this submission and believe that I have an appropriate level of expertise to state that I do not consider it to be of an acceptable scientific standard, for reasons outlined above.

# Response

**Published:** 2021-01-25

**Authors:** Friederike Kessel

**Affiliations:** University Hospital Carl Gustav Carus Dresden, Germany

We thank Prof. Molitoris for his time and the detailed review. However there seems to be a misunderstanding of the intentions of the submitted manuscript.

 We present and validate a workflow and an algorithm to process image data obtained from single nephron GFR measurements by intravital microscopy. The measurement itself is not the focus of the manuscript. A detailed protocol on animal preparation and image data acquisition was merely cited (1), however the experimental setup is reproducible using these protocols. In our setup, superficial glomeruli can be imaged in the intact kidney of 10-12 week old animals with 2-photon microscopy with sufficient quality. We want to specifically point out that removing parts of the kidney cortex prior imaging is not included in the cited protocols and we did not perform this in our study either. Prof. Molitoris is possibly referring to the paper of Kidokoro et al. (2) which was cited by us in the context of illustrating an existing analysis approach for image data obtained from the snGFR measurement.

 With the focus of the manuscript in mind: We compare results obtained from the analysis of the same image datasets with two different workflows (but using the same segments of the proximal tubule) – and not different experimental groups of differently treated animals. With all raw image data (openly accessible at Zenodo), the source code (openly accessible at GitHub) and open source software (ImageJ and R) the data we present are completely reproducible.

 We agree with Prof. Molitoris that presenting more image data in the manuscript might be beneficial. The challenges of depicting time series and complex 3D image data in a 2D representation led us to the decision to upload all raw image data (time series and 3D datasets) to Zenodo, where it can be freely accessed. Lastly we rephrase the description of one of the image processing steps: As Prof. Molitoris pointed out correctly, the subtraction of one z-stack from the other is performed plane by plane, and not with 3D volumes.

 Clarifying changes in the manuscript on the emphasis of the intention (presenting an image analysis workflow) will be made in an upcoming version. We would gladly invite Prof. Molitoris to review the updated manuscript again with this perspective.

1. Kidokoro K, Cherney DZI, Bozovic A, *et al.*: Evaluation of Glomerular Hemodynamic Function by Empagliflozin in Diabetic Mice Using In Vivo Imaging. *Circulation.* 2019; **140**(4): 303–315.
2. Schiessl IM, Fremter K, Burford JL, *et al.*: Long-Term Cell Fate Tracking of Individual Renal Cells Using Serial Intravital Microscopy. *Methods Mol Biol.* 2020; **2150**: 25–44.
